# Human ES Cell Culture Conditions Fail to Preserve the Mouse Epiblast State

**DOI:** 10.1155/2021/8818356

**Published:** 2021-03-10

**Authors:** A. S. Devika, Anna Montebaur, S. Saravanan, Raghu Bhushan, Frederic Koch, Smita Sudheer

**Affiliations:** ^1^Stem Cell Laboratory, Department of Genomic Science, Krishna Building, Central University of Kerala, Tejaswini Hills, Periye. P. O, Kasaragod District, Kerala 671316, India; ^2^Department of Developmental Genetics, Max Planck Institute for Molecular Genetics, Ihnestrasse 63-73, 14195 Berlin, Germany; ^3^Department of Biology, Chemistry and Pharmacy, Free University Berlin, Takustrasse 3, 14195 Berlin, Germany; ^4^Yenepoya (Deemed to be University), University Road, Deralakatte, Mangalore, 575018 Karnataka, India; ^5^Leibniz Institute for Farm Animal Biology (FBN), Institute for Genome Biology, Wilhelm-Stahl-Allee 2, D-18196 Dummerstorf, Germany

## Abstract

Mouse embryonic stem cells (mESCs) and mouse epiblast stem cells (mEpiSCs) are the pluripotent stem cells (PSCs), derived from the inner cell mass (ICM) of preimplantation embryos at embryonic day 3.5 (E3.5) and postimplantation embryos at E5.5-E7.5, respectively. Depending on their environment, PSCs can exist in the so-called naïve (ESCs) or primed (EpiSCs) states. Exposure to EpiSC or human ESC (hESC) culture condition can convert mESCs towards an EpiSC-like state. Here, we show that the undifferentiated epiblast state is however not stabilized in a sustained manner when exposing mESCs to hESC or EpiSC culture condition. Rather, prolonged exposure to EpiSC condition promotes a transition to a primitive streak- (PS-) like state via an unbiased epiblast-like intermediate. We show that the Brachyury-positive PS-like state is likely promoted by endogenous WNT signaling, highlighting a possible species difference between mouse epiblast-like stem cells and human Embryonic Stem Cells.

## 1. Introduction

Pluripotency is the intrinsic, unrestricted, flexible developmental potential of the embryonic cells in a developing embryo, to give rise to the three embryonic germ layers, ultimately forming all the cells in an adult organism. This can be captured *in vitro*, by deriving pluripotent stem cells (PSCs) from various developmental stages. The PSCs, derived from the epiblast of preimplantation mouse embryos (E3.75-E4.5) are called embryonic stem cells (ESCs) [1–3]. The mESCs can be brought to a so-called ground/naïve state of pluripotency, using leukaemia inhibitory factor (LIF) that sustains self-renewal [4, 5], in conjunction with the inhibition of ERK [2] and GSK3 [6] that simultaneously suppress differentiation (LIF/2i medium) [7]. The PSCs that are derived from the postimplantation embryos (E5.5-E7.5) are called the epiblast stem cells (EpiSCs), which are in a primed state of pluripotency [1, 8]. The mouse EpiSCs and human ESCs (hESCs) are conventionally cultured in Activin A and FGF2 (AA/F2). When the mESCs are exposed to the hESC/EpiSC condition (AA/F2), they transition to an EpiSC-like primed state [1, 8–11].

During gastrulation, the pluripotent epiblast cells in the developing mouse embryo undergo epithelial-mesenchymal transition (EMT) and migrate through the primitive streak (PS), forming mesendoderm cells, the common precursors of mesoderm and endoderm [12]. The epiblast cells that do not migrate through the PS form the neuroectoderm. Several signaling pathways play crucial roles in this rearrangement process, such as TGF*β*/activin/nodal, WNT/*β*-catenin, and FGF/ERK/MAPK signaling pathways [13–15]. The collaborative interaction between various transcription factors, such as the T-box transcription factors (Tbx) T and Eomes with the key markers of mesoderm and endoderm initiate this differentiation process [16–18].

Currently, a range of pluripotent states is being discovered [19, 20], and in spite of the fact that the EpiSCs share many properties [21] that safeguard their pluripotency, they possess unique functional and molecular properties that set them apart [22, 23]. This difference probably reflects on the transformations that the epiblast cells undergo, based on their spatiotemporal positions and their environment in the developing embryo, restricting their developmental potential to certain lineages. Under the currently known extrinsic culture conditions, the EpiSCs could possible occur in heterogeneous metastable states, possessing variable differentiation potentials [24, 25].

The standard hESC culture condition has been adapted to support mEpiSCs (Activin A and FGF2 (AA/F2)) [1, 8–11], and the same can convert mESCs in the ground/naïve state of pluripotency to a primed EpiSC-like state. This study is aimed at understanding the transition from naïve to primed state of pluripotency, under the standard hESC/EpiSC condition. In this study, we show that although this condition matured the mESCs to distinct primed EpiSC-like states, these states could not be sustained, but the cells further matured to a PS/mesendoderm-like state, highlighting a possible species difference between mouse and human epiblast-like stem cells. This also highlights the requirement of additional factors for the preservation of distinct pluripotency states of the mouse EpiSCs, in contrast to the requirement of hESCs that could enhance our capability to differentiate them into any desired cell type in high proportion.

## 2. Materials and Methods

### 2.1. Mouse ESC Lines

The experiments were carried out using the mouse embryonic stem cells (mESCs) from C57BL/6N background, having fluorescent reporters for Brachyury (T) and Sox2 [26]. Mouse bacterial artificial chromosomes (BACs) containing the part of the mouse genome comprising the locus of the specific gene (BACs RP24-530D23 (T) and RP23-249O15 (Sox2) containing ∼180-230 kb of the C57BL/6 mouse genome surrounding the respective loci), by Red/ET recombineering (GeneBridges) [27]. Shortly, the start codon (ATG) of T or Sox2 was replaced with mCherry or Venus coding sequence, followed by the rabbit b-globin polyadenylation signal and an FRT-site flanked hygromycin selection cassette, driven by the Pgk promoter. 5 mg of the modified BAC was linearized with the restriction enzyme PI-SceI (NEB) and electroporated into 3 × 10^6^ ESCs. After selection in ES cell medium containing hygromycin (150 mg/ml), clones were picked, expanded, and checked by PCR for BAC integration. For details, refer [26].

### 2.2. Cell Culture and Differentiation

ESCs and EpiSCs were cultured according to standard conditions [24]. For naïve conversion, the feeder-free mESCs were seeded on fibronectin- (3-5 ng/ml) (Merck-Millipore; Calbiochem) coated plates in N2B27 medium containing LIF, PD0325901 (1 *μ*M) (Axon Medchem, Axon 1408), and CHIR99021 (GSK3*β* inhibitor) (3 *μ*M) (Axon Medchem, Axon 1386) (2i medium) [7]. The next day, the cells were refed with the 2i medium. After the small dome-shaped colonies were seen, the medium was changed to N2B27 medium supplemented with Activin A (20 ng/ml) and FGF2 (10 ng/ml) (AA/F2). The AA/F2 treatment was continued for 6 days, while the medium is replaced with fresh AA/F2 medium every day.

### 2.3. Whole Transcriptome Data Analysis

RNA sequencing was carried out with 80 ng of total RNA. After quantification and quality assessment (details in [24]), the RNA-seq library was prepared from total RNA using the ScriptSeq Complete Kit (Human/Mouse/Rat)—Low Input (SCL24H) (Illumina), according to manufacturer's instructions. The prepared sequencing library was eventually sequenced on Illumina HiSeq 2000 for 2350 cycles (paired-end), following the standard protocol. The RNA-seq sequencing reads were mapped to the mouse genome (mm10) using TopHat (version 2.0.11) [28]. Cuffdiff was then used to calculate the normalized FPKM (fragments per kilobase per million mapped read) values for genes in all samples [29]. The results were filtered by removing genes with FPKM values lower than 1 in all samples, which were then used for further analysis (R statistical program) (http://www.r-project.org). The RNA-seq data has been deposited in the ArrayExpress database (E-MTAB-3784).

### 2.4. Real-Time PCR Analysis

Total RNA was isolated using RNeasy Micro and Mini Kits (Qiagen), followed by quantification using NanoDrop (Life Technologies) (unsorted cells) or Qubit fluorometer (Invitrogen) (sorted cells). The unsorted cells were directly lysed with the RLT buffer in the cell-culture wells, after rinsing once with PBS. An extra step of DNase I (Roche, Basel, Switzerland, https://www.roche-applied-science.com) treatment was carried out in order to avoid genomic DNA contamination. RNA was quantified using the spectrophotometer from either NanoDrop Technologies (unsorted cells) or Qubit (Invitrogen, Carlsbad, CA, http://www.invitrogen.com) (FACS-sorted cells). Reverse transcription was performed with the M-MLV reverse transcriptase (Promega) and Oligo-dT primers (Invitrogen) (Table S3). Quantitative reverse transcriptase PCR (qRT-PCR) was done using GoTaq qPCR Master Mix (Promega) with the help of validated gene-specific primers on StepOnePlus Real-Time PCR System (Life Technologies, Rockville, MD, https://www.lifetech.com). Data analysis was accomplished using the *ΔΔ*Ct method, with the housekeeping genes, *Pmm2* and *Gapdh* and/or *Actb* for normalization.

### 2.5. Fluorescence-Activated Cell Sorting (FACS)

The cells were gently rinsed with phosphate-buffered saline (PBS) and detached using ice cold trypsin. In a neutralization with cold knockout DMEM-F12, the cells were quickly (30 seconds) spun down at high speed (10,000 rpm) and resuspended in cold knockout DMEM-F12 and immediately placed on ice. The cells were then sorted on FACS Aria II (Becton Dickinson, Franklin Lakes, NJ, https://www.bd.com) directly into RLT buffer containing *β*-mercaptoethanol, followed by total RNA isolation using the RNeasy Micro Kit (Qiagen, Hilden, Germany, http://www1.qiagen.com) and processed further.

### 2.6. Immunoblotting and Immunocytochemistry

Standard procedures were followed for these assays (details in [24]) with the suitable antibodies (Table S4). The cells were initially rinsed with PBS, before immunoblotting and immunocytochemistry.

For immunoblotting, after adding the lysis buffer (containing protease inhibitor and phosphatase inhibitor (PhosStop, phosphatase inhibitor cocktail Easypack, Roche)), the cells were scraped off and lysed further, after placing on ice, to preserve the phosphorylation status of the proteins. Western blot data were quantified using ImageJ, a Java-based image analysis package. H3 and Actb served as controls.

For immunocytochemistry, the cells in monolayer culture were washed with PBS and fixed with 4% paraformaldehyde. After permeabilization, primary and secondary antibody staining were carried out. Visualization was done using a confocal microscope (LSM510 Meta, Zeiss), and further analysis was performed using the software, AxionVision (Zeiss).

## 3. Results

### 3.1. Human ESC/EpiSC Culture Condition Leads Naïve ESCs to Distinct EpiSC-Like States

**Figure 1:**
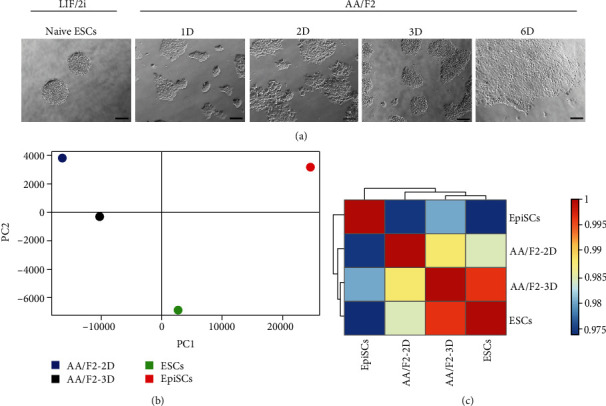
Human ESC/EpiSC culture condition leads naïve ESCs to distinct EpiSC-like states. (a) Brightfield images of the indicated samples (scale bars 100 *μ*m). mESCs were brought to naïve state with LIF/2i (LIF+2i (PD0325901+CHIR99021)), followed by AA/F2 treatment. This treatment results in morphological changes from round dome-shaped to flat colonies. (b) Principal component analysis (PCA) of the indicated samples. (c) Heat map, based on Pearson's correlation coefficient. AA/F2 treatment for different durations led naïve ESCs to primed EpiSC states that differed from each other and the PSCs that were compared here.

**Figure 2:**
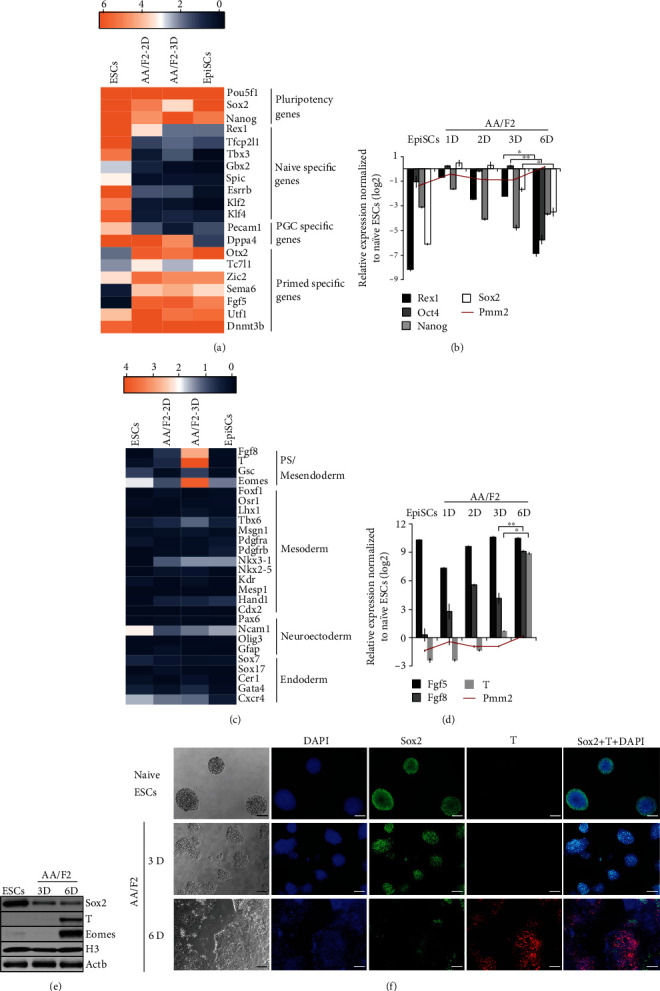
Human ESC/EpiSC culture condition matures the ESCs further to a primitive streak-like state. (a) Expression levels of the specific pluripotency-associated genes in the indicated samples. AA/F2 treatment led to the induction of primed pluripotency genes and downregulation of PGC and naïve pluripotency genes. (b) Analysis of pluripotency genes in AA/F2-treated cells in the indicated samples. *Gapdh* and *Pmm2*: housekeeping genes. *Rex1*, *Sox2*, and *Nanog* were downregulated, and *Oct4* expression was retained. Error bars: mean ± SD (*n* = 2); ^∗^*p* ≤ 0.05, ^∗∗^*p* ≤ 0.01, ^∗∗∗^*p* ≤ 0.001, and ^∗∗∗∗^*p* ≤ 0.0001 (*p* (Rex1) = 0.0107, *p*(Sox2) = 0.0421, and *p*(Oct4) = 0.0029) (Student's *t*-test). (c) Heat map showing the expression status of lineage-specific markers in the indicated samples in comparison with naïve mESCs. PS markers are induced in AA/F2-3 days (3D). (d) Analysis of EpiSCs (*Fgf5*) and PS markers (*T* and *Fgf8*) in the indicated samples. *Fgf5* and *Fgf8* were induced right from 1D, and by 6D, the PS marker *T* was also highly induced. Error bars: mean ± SD (*n* = 3); ^∗^*p* ≤ 0.05, ^∗∗^*p* ≤ 0.01, ^∗∗^*p* ≤ 0.001, and ^∗∗∗∗^*p* ≤ 0.0001 (*p*(Fgf8) = 0.0070 and *p*(T) = 0.0235) (Student's *t*-test). (e) Western blot analysis of the indicated markers in the indicated samples. Six days of AA/F2 treatment led to high protein expression of the PS/mesendoderm markers T and Eomes and downregulation of Sox2. (f) Representative immunostainings of the indicated proteins in the indicated samples (scale bars 100 *μ*m).

LIF/2i (LIF, PD0325901 (MEK inhibitor) and CHIR99021 (GSK3*β* inhibitor)), under defined conditions, can maintain mESCs in their naïve state of pluripotency [4, 5, 7] and AA/F2 (Activin A and FGF2) can transform them into a primed-like state [1, 8, 10]. A time course analysis for 3 days was performed to understand this transition of pluripotency states (Figure 1(a)). Round, dome-shaped colonies, characteristics of naïve ESCs, were observed when the mESCs were grown in LIF/2i. Upon AA/F2 exposure, mESCs gradually lost their dome-shaped morphology and grew as flat colonies. Expression of Rex1 is one of the hallmarks of the ground state [30]. Among the Oct4/Sox2/Nanog triumvirate, Oct4 and Sox2, defined as the core-pluripotency factors, are required for both the acquisition and maintenance of pluripotency [31], whereas Nanog is required only for the acquisition of pluripotency [32, 33]. AA/F2 treatment led to a decrease in the expression of *Rex1* and *Nanog* right from the first day, *S*ox2 from second day still retaining the expression of *Oct4* [31, 34, 35] till 3 days, showing the developmental advancement of the cells during the treatment (Figures 2(a), 2(b), 2(e), and 2(f), S1, Table S1). While Nanog expression is restricted exclusively to the nascent epiblast, Oct4 and Sox2 are ubiquitously expressed in the morula and blastocyst in all the cells of the inner cell mass (ICM), till the hypoblast segregates [36]. Whole transcriptome data revealed that the naïve-specific genes were downregulated, and the primed genes were induced during the treatment (Figures 2(a) and 2(b), Table S1). However, PCA and hierarchical clustering showed that the AA/F2-2D and AA/F2-3D cells were not only distinct from each other, but also from both the naïve ESCs and the EpiSCs (Figures 1(b)–1(d), S1, Table S1). This is a time when a spectrum of pluripotent states is being discovered [19, 20], and the mEpiSCs can be derived from E5.5 to E7.5 embryos that are similar in several molecular characteristics [21] and may also differ in the expression status of certain genes [22]. Taken together, our results demonstrate that the primed EpiSC-like states that were derived by the exposure of naïve ESCs to AA/F2 are distinct from each other and from the EpiSCs used in this study. The EpiSCs used here have been derived from early postimplantation E5.5 mouse embryos [9].

### 3.2. Human ESC/EpiSC Culture Condition Matures the ESCs Further to Primitive Streak-Like (PS-Like) State

The changes in pluripotency genes by three days coincided with the induction of *T*, *Eomes*, and *Fgf8*, and the genes were expressed in the PS (Figures 2(a)–2(e), Table S1). At protein level, the induction of T was low, which could be attributed to a probable delay between transcription and translation. At this point (AA/F2-3D), no significant induction of any of the lineage-specific markers, mesoderm (Foxf1, Gata6, Osr1, Tbx6, and Msgn1), endoderm (Sox17), neuroectoderm (Pax6 and Sox1), or primitive endoderm (Sox7) had occurred (Figure 2(c), Table S1). The EpiSCs derived from late postimplantation embryos (E7.5) have reduced neural induction potential and express T and OCT4 [22]. Therefore, AA/F2 treatment for 3 days matured ESCs from the naïve pluripotency to a more matured primed EpiSC-like state.

EpiSCs are routinely maintained using AA/F2 [1]. To know if this condition will maintain the converted EpiSC-like cells, the AA/F2 treatment was continued for 6 days. This led to the downregulation of the core pluripotency factors, *Oct4*, *Sox2*, and *Nanog* (Figures 2(b), 2(d), and 2(e), Table S1), and significant induction of the PS genes, *Fgf8* and *T* (Figures 2(c)–2(e)). Taken together, although the EpiSC culture condition matures the naïve ESCs to a primed-like state, the continuous exposure of this condition for six days transforms these cells into a PS-like or mesendoderm-like state.

### 3.3. Epithelial-Mesenchymal Transition Occurs during Maturation to the PS-Like State

**Figure 3:**
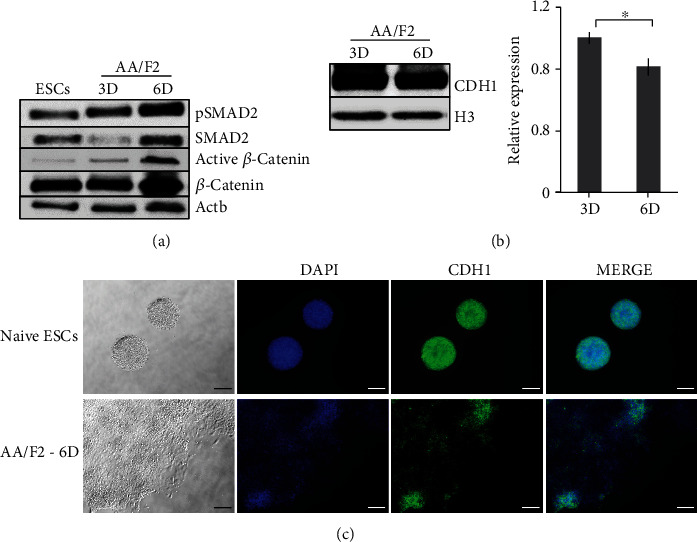
Epithelial-mesenchymal transition occurs during maturation to the PS-like state. (a) Western Blot analysis of the markers involved in WNT and TGF*β* signaling pathways in the indicated samples (relative quantification graph: Figure S2A). Activin/nodal (pSMAD2) and WNT (active *β*-catenin) signaling were highly active by 6 days of AA/F2 treatment, compared to 3 days. Actb: control. (b) Western blot analysis of the epithelial marker CDH1 protein in the indicated samples. Histone H3 (used for normalization) and Actb (Figure S2D) served as controls. There was a slight decrease in the expression of CDH1. Error bars: mean ± SD (*n* = 2); ^∗^*p* ≤ 0.05; *p*(CDH1) = 0.0471 (Student's *t*-test). (c) Representative immunostainings of the indicated proteins in the cells treated with AA/F2 for 6 days (scale bars 100 *μ*m).

Epithelial-mesenchymal transition (EMT) and the migration of pluripotent epiblast cells through the PS facilitate the formation of mesoderm and endoderm progenitors [12]. Brachyury (T) is required for EMT and PS induction [37, 38] and is stimulated by the collaborative cross talk between FGF and WNT signaling [24, 39]. *β*-Catenin regulates PS induction by promoting SMAD2/3 activity [39, 40]. By 6 days of AA/F2 exposure, T, Eomes, and *Fgf8* were highly induced (Figures 2(c)–2(e)), when SMAD2 was phosphorylated and *β*-catenin phosphorylation inhibited (Figure 3(a), S2A), reflecting on the active status of TGF*β*/activin/nodal and canonical WNT signaling pathways. This coincided with a modest downregulation of the epithelial marker E-cadherin (CDH1) (Figures 3(b) and 3(c), S2D). We also found higher induction of AKT and total ERK in AA/F2-6D cells (Figure S2B-S2C). The epithelial cells in the epiblast express CDH1 which is repressed during PS formation, which in turn induces EMT [41]. Overall, the continued EpiSC condition for 6 days promotes EMT, a crucial process that occurs during PS formation.

### 3.4. Continuous Exposure to the hESC/EpiSC Condition Draws the PSCs towards Posterior Mesoderm and Endoderm Lineages

**Figure 4:**
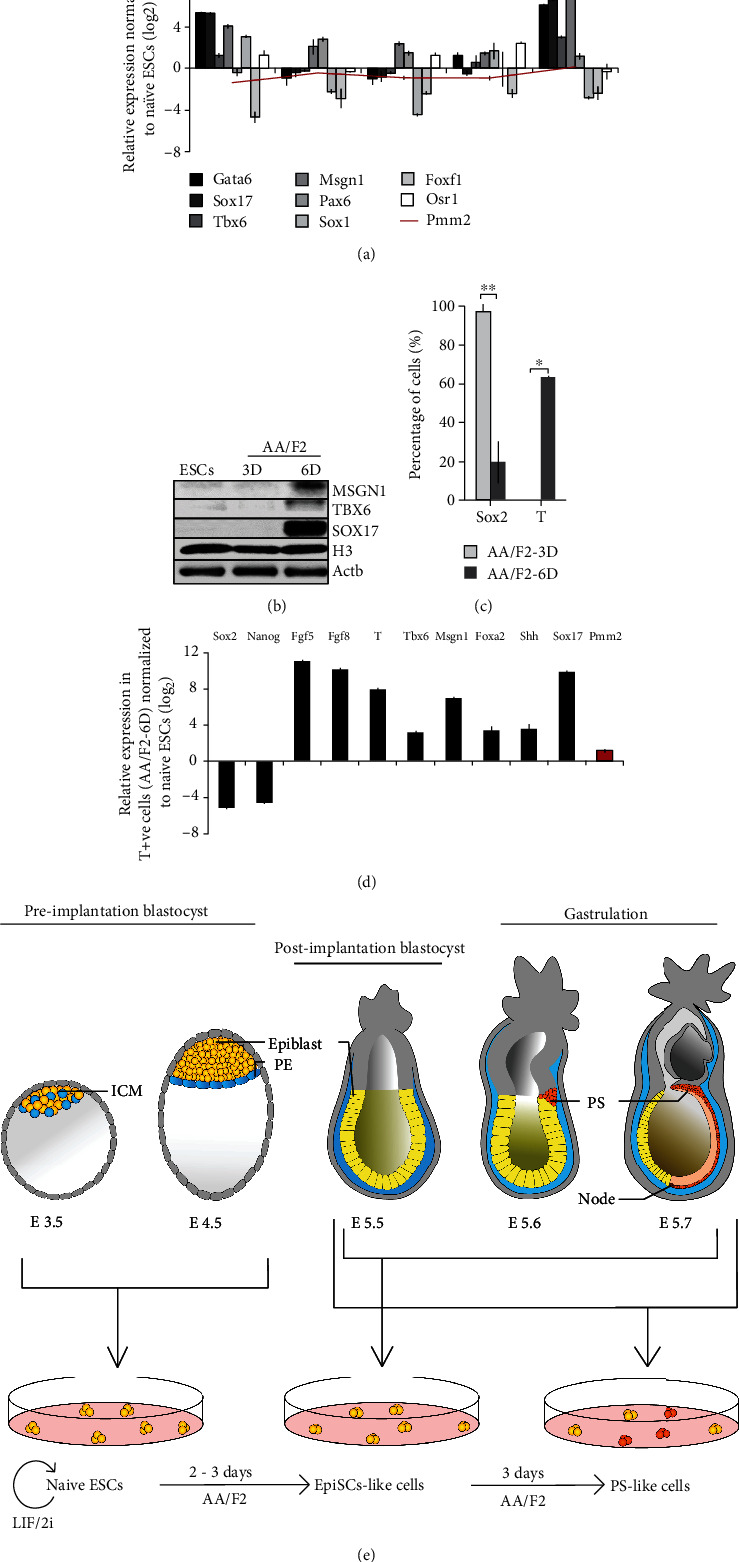
Continuous exposure to the hESC/EpiSC condition draws the PSCs towards posterior mesoderm and endoderm lineages. (a) qRT-PCR analysis of the indicated lineage-specific genes in the indicated samples. *Gapdh* and *Pmm2:* housekeeping genes. The endoderm (*Gata6* and *Sox17*) and posterior mesoderm (*Tbx6* and *Msgn1*) markers were induced by 6 days. The neuroectoderm (*Pax6* and *Sox1*), lateral mesoderm (*Foxf1*), and intermediate mesoderm (*Osr1*) genes were not expressed. (b) Western blot analysis of the mesoderm (Msgn1 and Tbx6) and endoderm (Sox17) proteins in the cells treated with AA/F2 for the indicated durations. The posterior mesoderm (Msgn1 and Tbx6) and endoderm (Sox17) proteins were expressed by 6 days of the treatment. (c) Flow-cytometric analysis of the fraction of *Sox2*+ve and *T*+ve cells after 3 and 6 days of AA/F2 treatment. Error bars: mean ± SD (*n* = 2); ^∗^*p* ≤ 0.05 and ^∗∗^*p* ≤ 0.01 (*p*(Sox2) = 0.0070 and *p*(T) = 0.0098) (Student's *t*-test). By 6 days of treatment, the count of *Sox2*-expressing cells was very low, and almost half the proportion of the cells expressed the PS marker, *T*. (d) Analysis of the indicated genes in the FACS-sorted *T*+ve cells from the AA/F2-6D-treated cells. The *T*-expressing cells expressed early markers of PS, mesoderm, and endoderm. (e) A model that summarizes the major finding of this study. The mESCs were converted to naïve state of pluripotency (represent the pluripotent embryonic cells in the E3.5-E4.5 developmental stages) using the 2i medium (LIF/2i). AA/F2 treatment for 2 to 3 days led these cells to a primed state of pluripotency (may represent the pluripotent epiblast cells in the E5.5-E7.5 developmental stages). This study shows that the continued treatment of AA/F2 for 6 days led to the enrichment of cells expressing the genes of the primitive streak, mesoderm (primarily, posterior mesoderm), and endoderm (may represent the pluripotent epiblast cells and PS/mesendoderm cells in the E6.5-E7.5 developmental stages).

During early development, the cardiac mesoderm and endoderm arise from the anterior PS, whereas somitic and extraembryonic mesoderm originate from the posterior PS [42, 43]. AA/F2 treatment for 6 days resulted in the upregulation of endoderm (Sox17 and *Gata6*) and posterior mesoderm markers (Tbx6 and Msgn1) (Figures 4(a) and 4(b), S3), reflecting on a mixed population of cells. In the epiblast, while high activin/nodal signaling supports endoderm differentiation [18, 44], WNT/*β*-catenin supports posterior PS genes [45]. Both these pathways were active in the AA/F2-6D cells (Figure 3(a)), and at this stage, T was highly expressed (Figures 2(c)–2(e)), with considerable population of the cells expressing *T* (Figure 4(c)). However, the neuronal (Sox2, *Pax6*, and *Sox1*), lateral mesoderm (*Foxf1*), and the intermediate mesoderm (*Osr1*) (Figure 4(a)) genes were not noticeable expressed then. Endogenous WNT signaling in EpiSCs induces loss of pluripotency, with increase in the proportion of T-positive cells and differentiation tendency towards definitive endoderm [46]. Analysis of the sorted *T*-positive cells revealed that they expressed the epiblast (*Fgf5*), the posterior mesoderm (*Fgf8*, *T*, *Msgn1*, and *Tbx6*), and the endoderm (*Foxa2* and *Sox17*) markers (Figure 4(d)). The pluripotency markers, *Nanog* and *Sox2*, were downregulated. In contrast, majority of the AA/F2-3D cells that were in the EpiSC-like state expressed *Sox2* and there were no *T*-positive cells among them. This highlights the importance of early passaging of EpiSCs that might be able to avoid their further maturation towards PS/mesendoderm-like state in order to establish stable cell lines. These observations summarize that the continuous AA/F2 treatment leads the naïve PSCs to a primed EpiSC-like state, followed by PS- or mesendoderm-like state, supporting the endoderm and posterior mesoderm lineages (Figure 4(e)).

## 4. Discussion

During embryo development, cells in the epiblast undergo several systematic changes in pluripotency and part of it can be mimicked *in vitro*. Mouse ESCs can be brought to a ground/naive state of pluripotency using LIF, along with the inhibitors of ERK and GSK3 [7] (2i medium). The standard hESC culture condition has been adapted to support mEpiSCs (Activin A and FGF2 (AA/F2)), and the same can convert mESCs in the ground/naïve state of pluripotency to a primed EpiSC-like state [1, 8–11]. The current study demonstrates that the exposure of mESCs in the ground state of pluripotency to the AA/F2 transitions them through distinct primed-like EpiSC states, and prolonged exposure of this condition leads them to a PS-like state that had active WNT and activin/nodal signaling and expressed T, Eomes, posterior mesoderm, and endoderm markers (Figure 4(e)). They did not express neuronal markers.

T and Eomes together promote the upregulation of posterior mesoderm and endoderm markers [18, 47] and are induced by FGF, WNT/*β*-catenin, and activin/nodal/TGF*β* signaling, with a negative impact on pluripotency and neural markers [9, 18]. Moreover, the endogenous WNT signaling in EpiSCs under hESC culture condition which causes loss of pluripotency and WNT inhibition could prevent this, reflecting on possible species-specific difference in the culture conditions of mouse and human [46, 48–50]. Although the hESC/EpiSC condition (AA/F2) matured the mESCs to primed states, we show that these states could not be sustained, but the cells further matured to a PS/mesendoderm-like state that had higher WNT signaling active, compared to the intermediate EpiSC-like states (AA/F2-2D and AA/F2-3D). This highlights the requirement of additional factors such as WNT inhibitors, for the preservation of primed pluripotent states of the mouse EpiSCs, in contrast to the requirement of human ESCs [46, 48–50].

Although the AA/F2-treated cells showed primed-like features by both 2 and 3 days of induction, they were different from each other and also from the EpiSCs derived from early postimplantation stage (E5.5) [9]. The 3-day AA/F2-matured EpiSC-like cells, in addition to the pluripotency genes (*Oct4*, *Sox2*, *and Nanog*), also expressed *T*, *Eomes*, and *Gsc.* These characteristics resemble the features of the EpiSCs derived from late postimplantation embryos that express T [22]. The EpiSCs can be derived from mouse postimplantation embryos at various embryonic stages (E5.5–E7.5) that in spite of being similar in several primed-state characteristics [21], also differ among each other [22]. The emerging theory from recent studies is that among pluripotent or progenitor cells in the epiblast, there could be a wide range of molecular states having specific underlying molecular signatures. This heterogeneous population of epiblast cells having diverse differentiation capacities could depend on slightly different extrinsic culture conditions for maintenance that confer their pluripotent or multipotent properties [51], and a majority of this information still remains undefined. For instance, the different durations of AA/F2 led ESCs to distinct pluripotent states that yielded different proportions of presomitic mesoderm cells [24]. At present, the naïve and primed states of pluripotency are the only well-defined states in human and mouse that are interconvertible and can be maintained under defined *in vitro* conditions. The *in vitro* conditions used to maintain ESCs could influence the gene-expression state and cell populations within the culture largely [52, 53]. Different populations of EpiSCs, distinct from ESCs, that are interconvertible *in vitro*, such as Brachyury or Oct4 positive or negative, can coexist, whereby the *Oct4*+ve cells still can retain the chimera-forming ability [54, 55], which shows that they are totipotent. Although there have been attempts to capture totipotency *in vitro* [56, 57], the exact conditions that maintain such a state are still unknown [58]. Under the currently known extrinsic culture conditions, possibly, the EpiSCs could occur in heterogeneous metastable states, possessing variable differentiation potentials [24, 25], even to extraembryonic lineages, such as trophoblast [1, 8, 59–62]; [63–66]. The different states could be preserved or interconvertible with ease, by mere manipulation of signaling pathways. The discovery of the 3i: (PD184353, PD173074/SU5402, and CHIR99021, respectively) and later 2i (CHIR99021 to inhibit GSK3*β* and PD0325901 to inhibit MEK1/2) media by Austin Smith and colleagues (Ying et al. 2008) increased the ESC derivation efficiency in nonpermissive mouse and rat strains, such as CBA, NOD, and DBA from low or nonexistent to 50–70% [67–73]. Therefore, it is very crucial to explore the possibilities of maintaining specific states of totipotency or pluripotency in culture, not only to further increase the efficiency of ESC derivation from any strain and chimera-forming ability, but also to facilitate studies on placental and early embryonic development. More investigation into the specific factors required for maintaining a particular totipotency or pluripotency state could enhance our capability to differentiate them into any desired cell type more efficiently, in high proportion, and our understanding of mammalian embryo development. This and further research will be vital for understanding the molecular mechanism of early patterning in embryogenesis and besides, will ensure that knowledge will be gained about totipotency, pluripotency, and differentiation into progenitor cells for scientific purpose as well as regenerative medicine and thereby associated stem cell-based therapies.

## Data Availability

The data that support the findings of this study are available from the corresponding author, upon reasonable request.

## Supplementary Materials

Supplementary Materials Figure S1: human ESC/EpiSC culture condition leads naïve ESCs to distinct EpiSC-like states. (a) Hierarchical clustering of the indicated samples. The hierarchical clustering indicates that the AA/F2-treated cells were distinct from both EpiSCs (from early postimplantation E5.5 mouse embryos) and ESCs. Figure S2: epithelial-mesenchymal transition occurs during maturation to the PS-like state. (a) Western blot analysis (quantification of the Western blot image in Figure 3(a)) of the indicated proteins in the indicated samples. The graph indicates the relative expression of the indicated proteins in AA/F2-6D, with respect to their expression in AA/F2-3D. Activin/nodal (pSMAD2) and WNT (active *β*-catenin) signaling were highly active by 6 days of AA/F2 treatment, compared to 3 days. (b–d). Western blot analysis of the indicated proteins in the cells treated with AA/F2 for the indicated durations. Actb served as control. The expression of total AKT and ERK increased by 6 days. There was a slight decrease in the expression of CDH1. Actb and H3 (Figure 3(b)) served as controls. Figure S3: Continuous exposure to the hESC/EpiSC condition draws the PSCs towards of posterior mesoderm. Analysis of the indicated genes in the cells treated with AA/F2 for the indicated durations. The posterior mesoderm marker Msgn1 was induced by 6 days of treatment, and its expressions were negligible at earlier states. Table S1: AA/F2 treatment for three days results in the downregulation of pluripotency genes and the expression of primitive streak genes. FPKM (log2) values of pluripotency and lineage-specific genes (Figures 1(b) and 2(a)). Table S2: Pearson's correlation coefficients between the samples (Figure 1(d)). Table S3: list of primers and their sequences (5′-3′) used for qRT-PCR. Table S4: list of primary and secondary antibodies used for Western blots and immunocytochemistry.
