# Supplementary material for: Human ES Cell Culture Conditions Fail to Preserve the Mouse Epiblast State
Source: Stem Cells Int. 2021 Mar 10;2021:8818356. doi: 10.1155/2021/8818356 (PMC8004371; doi:10.1155/2021/8818356)
Supplement: Supplementary Materials — Figure S1: human ESC/EpiSC culture condition leads naïve ESCs to distinct EpiSC-like states. (a) Hierarchical clustering of the indicated samples. The hierarchical clustering indicates that the AA/F2-treated cells were distinct from both EpiSCs (from early postimplantation E5.5 mouse embryos) and ESCs. Figure S2: epithelial-mesenchymal transition occurs during maturation to the PS-like state. (a) Western blot analysis (quantification of the Western blot image in Figure 3(a)) of the indicated proteins in the indicated samples. The graph indicates the relative expression of the indicated proteins in AA/F2-6D, with respect to their expression in AA/F2-3D. Activin/nodal (pSMAD2) and WNT (active β-catenin) signaling were highly active by 6 days of AA/F2 treatment, compared to 3 days. (b–d). Western blot analysis of the indicated proteins in the cells treated with AA/F2 for the indicated durations. Actb served as control. The expression of total AKT and ERK increased by 6 days. There was a slight decrease in the expression of CDH1. Actb and H3 (Figure 3(b)) served as controls. Figure S3: Continuous exposure to the hESC/EpiSC condition draws the PSCs towards of posterior mesoderm. Analysis of the indicated genes in the cells treated with AA/F2 for the indicated durations. The posterior mesoderm marker Msgn1 was induced by 6 days of treatment, and its expressions were negligible at earlier states. Table S1: AA/F2 treatment for three days results in the downregulation of pluripotency genes and the expression of primitive streak genes. FPKM (log2) values of pluripotency and lineage-specific genes (Figures 1(b) and 2(a)). Table S2: Pearson's correlation coefficients between the samples (Figure 1(d)). Table S3: list of primers and their sequences (5′-3′) used for qRT-PCR. Table S4: list of primary and secondary antibodies used for Western blots and immunocytochemistry. [file 8818356.f1.docx]

**Supplemental information**

**Human ES cell culture conditions fail to preserve the mouse epiblast state**

**Devika A. S, Anna Montebaur, Saravanan S, Raghu Bhushan, Frederic Koch, Smita Sudheer**

**Supplemental Figures:**


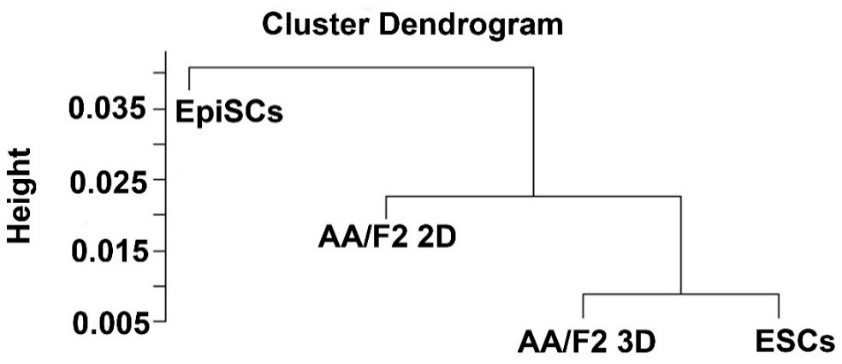


**Figure S1. Human ESC/EpiSC culture condition lead naïve ESCs to distinct EpiSC-like states.** **A.** Hierarchical clustering of the indicated samples. The hierarchical clustering indicates that the AA/F2 treated cells were distinct from both EpiSCs (from early post-implantation E5.5 mouse embryos) and ESCs.


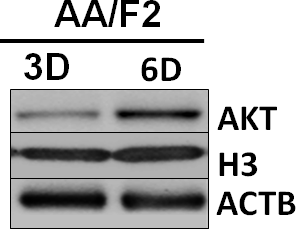

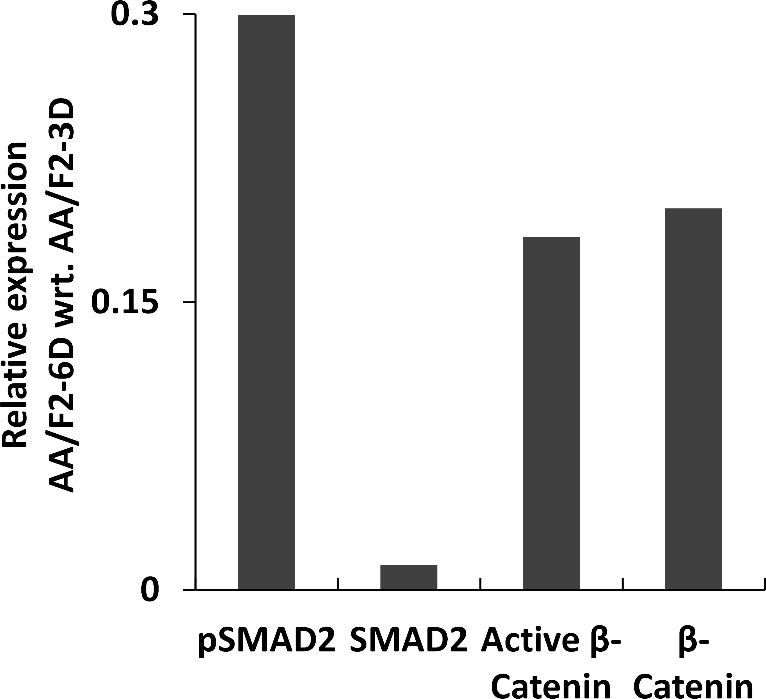


**B**

**A**


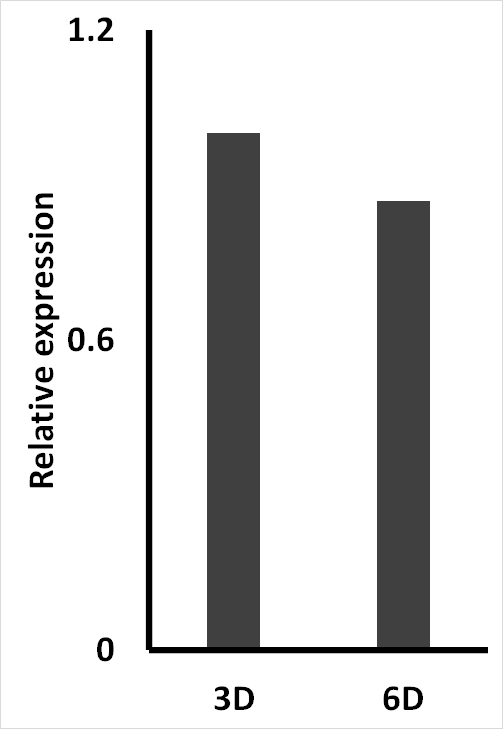


**C**

**D**


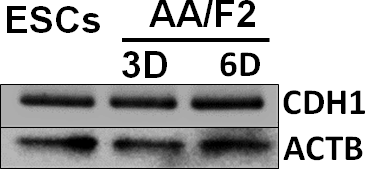

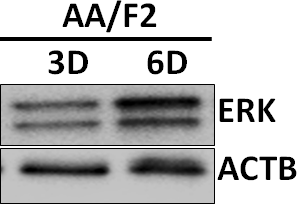


**Figure S2**. **Epithelial-mesenchymal transition occurs during maturation to the PS-like state. A.** Western blot analysis (quantification of the western blot image in Figure 3A) of the indicated proteins in the indicated samples. The graph indicates the relative expression of the indicated proteins in AA/F2-6D, with respect to their expression in AA/F2-3D. ACTIVIN/NODAL (pSMAD2) and WNT (Active β-Catenin) signalling were highly active by 6 days of AA/F2 treatment, compared to 3 days. **B, C & D.** Western Blot analysis of the indicated proteins in the cells treated with AA/F2 for the indicated durations. ACTB served as control. The expression of Total AKT and ERK increased by 6 days. There was a slight decrease in the expression of CDH1. ACTB and H3 (Figure 3B) served as controls.


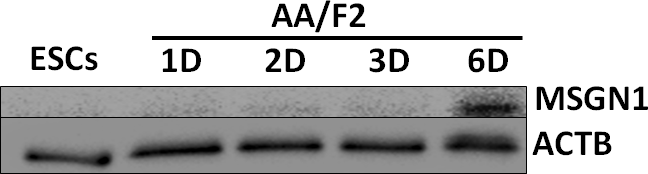


**Figure S3.** **Continuous exposure to the hESC/EpiSC condition draws the PSCs towards of posterior mesoderm.** Analysis of the indicated genes, in the cells treated with AA/F2 for the indicated durations. The posterior mesoderm marker MSGN1 was induced by 6 days of treatment and its expressions was negligible at earlier states.

**Supplemental Tables:**

| Gene groups | Genes | Naïve ESCs | AA/F2_2D | AA/F2_3D | EpiSCs |
| --- | --- | --- | --- | --- | --- |
| Pluripotency genes | *Pou5f1 (Oct4)* | 8.538712 | 8.562663 | 7.827552 | 7.656763 |
|  | *Sox2* | 8.06471 | 4.964085 | 3.478776 | 6.428902 |
|  | *Nanog* | 8.653923 | 4.59018 | 5.940788 | 5.028915 |
| Naïve-specific genes | *Tfcp2l1* | 7.414592 | 1.395908 | 1.83156 | 1.414745 |
|  | *Tbx3* | 5.087531 | 0.588724 | 1.637365 | 0.031596 |
|  | *Gbx2* | 2.668814 | 0.824122 | 0.19648 | 0.137586 |
|  | *Spic* | 3.205511 | 0.354576 | 0.449399 | 0.436656 |
|  | *Esrrb* | 6.479709 | 1.617185 | 1.538223 | 0.292213 |
|  | *Rex1* | 6.735956 | 3.404709 | 1.921833 | 1.900786 |
|  | *Klf2* | 4.790386 | 0.558427 | 0.430617 | 0.07742 |
|  | *Klf4* | 5.622538 | 0.617093 | 0.891649 | 0.667096 |
|  | *Gbx2* | 2.668814 | 0.824122 | 0.19648 | 0.137586 |
| PGC-specific genes | *Pecam1* | 3.835963 | 1.663244 | 0.570795 | 1.534123 |
|  | *Dppa4* | 6.468009 | 6.771361 | 4.740472 | 1.324564 |
| Primed-specific genes | *Otx2* | 1.900978 | 5.68311 | 5.179686 | 5.974708 |
|  | *Tcf7l1* | 2.230111 | 3.258212 | 2.517432 | 2.978046 |
|  | *Zic2* | 3.394881 | 5.283557 | 4.742587 | 4.728617 |
|  | *Sema6a* | 0.646649 | 3.751809 | 4.148668 | 3.491337 |
|  | *Fgf5* | 0.271513 | 5.576128 | 5.670595 | 4.88228 |
|  | *Utf1* | 3.869084 | 6.491584 | 5.170733 | 5.360447 |
|  | *Dnmt3b* | 5.568262 | 9.418906 | 7.889869 | 6.912843 |
|  | *Fgf5* | 0.271513 | 5.576128 | 5.670595 | 4.88228 |
| PS/Mesendoderm genes | *Fgf8* | 0 | 0.737716 | 2.809389 | 0.154632 |
|  | *T* | 0.223772 | 0.629862 | 3.966743 | 0.102063 |
|  | *Gsc* | 0.883141 | 0.152262 | 0.884702 | 0.11169 |
|  | *Eomes* | 1.903863 | 1.026443 | 3.806631 | 1.351669 |
| Mesoderm genes | *Foxf1* | 0 | 0.215763 | 0.02782 | 0.169911 |
|  | *Osr1* | 0.123619 | 0.097683 | 0.152892 | 0.166016 |
|  | *Lhx1* | 0 | 0.073995 | 0.283775 | 0.154145 |
|  | *Tbx6* | 0.374121 | 0.628887 | 1.213434 | 0.4997 |
|  | *Msgn1* | 0.141114 | 0.200383 | 0.323976 | 0.182296 |
|  | *Pdgfra* | 0.332788 | 0.104662 | 0.172666 | 0.329917 |
|  | *Pdgfrb* | 0 | 0.154581 | 0.173005 | 0.47115 |
|  | *Nkx3-1* | 0.028939 | 1.101552 | 1.474983 | 1.483021 |
|  | *Nkx2-5* | 0.050153 | 0.288771 | 0.303374 | 0.07812 |
|  | *Kdr* | 0.241649 | 0.049386 | 0.198005 | 0.036538 |
|  | *Mesp1* | 0 | 0 | 0 | 0 |
|  | *Hand1* | 0.046025 | 0.508994 | 0.56225 | 0.718837 |
|  | *Cdx2* | 0 | 0.017858 | 0.073979 | 0 |
| Neuroectoderm genes | *Pax6* | 0.037189 | 0.121726 | 0.06927 | 0.073404 |
|  | *Ncam1* | 2.125789 | 0.981028 | 1.315031 | 1.568123 |
|  | *Olig3* | 0 | 0 | 0.047521 | 0.067324 |
|  | *Gfap* | 0.054319 | 0.222697 | 0.079167 | 0.034413 |
| Endoderm genes | *Sox7* | 0.566483 | 0.299306 | 0.203515 | 0.226274 |
|  | *Sox17* | 0 | 0.215019 | 0.08454 | 0 |
|  | *Cer1* | 0.200259 | 0.023176 | 0.236173 | 0.041631 |
|  | *Gata4* | 0.499695 | 0.265039 | 0.641389 | 0.125845 |
|  | *Cxcr4* | 1.662766 | 1.310182 | 1.236003 | 0.419983 |

**Table S1. AA/F2 treatment for three days results in the down-regulation of pluripotency genes and the expression of primitive streak genes.** FPKM (log_2_) values of Pluripotency and lineage-specific genes (Figure 1B, 2A)

|  | **AA_fgf2_2D** | **AA_fgf2_3D** | **Naïve ESCs** | **EpiSCs** |
| --- | --- | --- | --- | --- |
| **AA/FGF2-2D** | 1 | 0.98867 | 0.98498 | 0.97511 |
| **AA/FGF2-3D** | 0.98867 | 1 | 0.99651 | 0.98067 |
| **Naïve ESCs** | 0.98498 | 0.99651 | 1 | 0.97429 |
| **EpiSCs** | 0.97511 | 0.98067 | 0.97429 | 1 |

**Table S2.** Pearson’s correlation coefficients between the samples (Figure 1D)

| **Primer** | **Sequence (5’- 3’)** |
| --- | --- |
| **Housekeeping genes** | |
| Gapdh (fwd) | CCA ATG TGT CCG TCG TGG AT |
| Gapdh (rev) | TGC CTG CTT CAC CAC CTT CT |
| Pmm2 (fwd) | AGG GAA AGG CCT CAC GTT CT |
| Pmm2 (rev) | AAT ACC GCT TAT CCC ATC CTT CA |
| **Naïve specific genes** | |
| Rex1 (fwd) | GGC TGC GAG AAG AGC TTT ATT CA |
| Rex1 (rev) | AGC ATT TCT TCC CGG CCT TT |
| **Primed specific genes** | |
| Fgf5 (fwd) | CCT TGC GAC CCA GGA GCT TA |
| Fgf5 (rev) | CCG TCT GTG GTT TCT GTT GAG G |
| **Pluripotency associated genes** | |
| Oct4 (fwd) | TGT TCC CGT CAC TGT TCT GG |
| Oct4 (rev) | TTG CCT TGG CTC ACA GCA TC |
| Sox2 (fwd) | AGA CCG TTT TCG TGG TCT TG |
| Sox2 (rev) | TAT CAA CCT GCA TGG GCA TT |
| Nanog (fwd) | GAA CGG CCA GCC TTG GGA T |
| Nanog (rev) | GCA ACT GTA CGT AAG GCT GCA GAA |
| **EMT specific genes** | |
| Cdh1 (fwd) | CCT GCC AAT CCT GAT GAA AA |
| Cdh1 (rev) | GAA CCA CTG CCC TCG TAA TC |
| **PS/ Mesendoderm specific genes** | |
| Fgf8 (fwd) | TCG CGA AGC TCA TTG TGG A |
| Fgf8 (rev) | GCC GTTG CTC TTG GCA ATT AG |
| T (fwd) | TTG AAC TTT CCT CCA TGT GCT GA |
| T (rev) | TCC CAA GAG CCT GCC ACT TT |
| **Mesoderm specific genes** | |
| Msgn1 (fwd) | GCC TGG ACT CTT CTG ACA CC |
| Msgn1 (rev) | TAG GAC TCC AGA GAA GGA GCT G |
| Tbx6 (fwd) | GGT AGC ATC CGC ATT GAA GT |
| Tbx6 (rev) | CTG AGC TTT GAA GCC AGA GG |
| Foxf1 (fwd) | CAA CGC ATC CCT CGG TAT CAC |
| Foxf1 ( rev) | GAG GCC ATG GCA TTG AAA GAG |
| Osr1 (fwd) | TTT CCA GTC CCC CTT CCT TTC |
| Osr1 (rev) | CCG GAT GGC AGA AGC AGA TAC |
| **Neuroectoderm specific genes** | |
| Sox1 (fwd) | GGC CGA GTG GAA GGT CAT GT |
| Sox1 (rev) | TCC GGG TGT TCC TTC ATG TG |
| Snai1 (fwd) | CAC CCT CAT CTG GGA CTC TC |
| Snai1 (rev) | ACA GCG AGG TCA GCT CTA CG |
| Shh (fwd) | TGT ACG TGG TGG CTG AAC G |
| Shh (rev) | GGT TGA TGA GAA TGG TGC CG |
| **Endoderm specific genes** | |
| Foxa2 (fwd) | ACA ACC TCA TGT CGT CCG AG |
| Foxa2 (rev) | CCT GGG TAG TGC ATG ACC TG |
| Gata6 (fwd) | CGG TTA TCC CAG AAC CCA TTC |
| Gata6 (rev) | CAC TCC GCC TCC AGG ATA GAC |
| Sox17 (fwd) | AGC CAT TTC CTC CGT GGT GT |
| Sox17 (rev) | AAC ACT GCT TCT GGC CCT CAG |

**Table S3: List of primers and their sequences (5’- 3’) used for qRT PCR**

| **Antibody** | **Details** |
| --- | --- |
| **Primary antibodies** | |
| Active- β-CATENIN | Monoclonal, mouse IgG  Millipore 05-665 |
| CDH1 | Monoclonal, mouse IgG  BD 610182 |
| MSGN1 | Polyclonal, homemade, affinity purified, rabbit IgG  Department Prof. Herrmann, MPIMG, Berlin |
| pSMAD2 | Polyclonal, rabbit IgG  Cell signaling 3101S |
| SMAD2 | Polyclonal, rabbit IgG  Cell signaling 3102 |
| SOX17 | Polyclonal, goat IgG  BD AF 1924 |
| SOX2 | Polyclonal, mouse IgG  Abcam ab79351 |
| T | Polyclonal, homemade, affinity purified, rabbit IgG (Department Prof. Herrmann, MPIMG, Berlin) |
| TBX6 | Polyclonal, rabbit IgG  Abcam ab38883 |
| ACTB | Monoclonal, mouse IgG1  Sigma-Aldrich A5441 |
| β-CATENIN | Monoclonal, mouse IgG  BD 610153 |
| AKT | Rabbit polyclonal  Cell Signalling Technology 9272 |
| HistoneH3 | H3 (abcam: ab1791) |
| ERK | Erk (cell signaling: CS #9102) |
| **Secondary antibodies** | |
| Anti-rabbit | IgG, HRP-linked Antibody  cell signaling 7074S |
| Anti-mouse | IgG, HRP-linked Antibody  Cell signaling 7076S |
| Anti-goat | IgG, HRP-linked Antibody  Immunoresearch, Jackson 705036147-70686 |

| **Table S4: List of primary and secondary antibodies used for western blots and Immunocytochemistry** |  |  |
| --- | --- | --- |
|  |  |  |
